# Adaptive Convolutional Neural Network-Enhanced Scale-Fusion Network for Human Activity Recognition Using Wearable Sensors

**DOI:** 10.3390/s26185879

**Published:** 2026-09-17

**Authors:** Dongpeng Xie, Peihui Yan, Yifei Li, Qinghai Wang, Jingnan Liu

**Affiliations:** 1 GNSS Research Center, Wuhan University, Wuhan 430079, China; dongpengxie@whu.edu.cn (D.X.); yfeili@whu.edu.cn (Y.L.); jnliu@whu.edu.cn (J.L.); 2 Hubei Luojia Laboratory, Wuhan 430079, China; wangqinghai@whu.edu.cn; 3 University Student Engineering Training and Innovation Practice Center, Wuhan University, Wuhan 430072, China

**Keywords:** adaptive kernel convolution, attention mechanism, convolutional neural network, deep learning, human activity recognition, sensor

## Abstract

Deep learning has achieved notable success in sensor-based human activity recognition (SHAR), yet wearable inertial signals contain activity patterns at different temporal scales while practical deployment imposes strict computational constraints. This paper proposes a three-stage Adaptive CNN-Enhanced Scale-fusion Network (ACESNet) for six-channel accelerometer–gyroscope activity recognition. The Adaptive Kernel Encoder dynamically combines temporal kernels of different sizes, while the CNN-Enhanced Scale-fusion design combines multi-scale temporal mixing, explicit channel mixing and a parallel local CNN path without materializing a global attention matrix. The main benchmark uses a fixed stratified 70/15/15 window-level partition (split seed 42), and all methods in the main comparison are repeated over five training seeds. On REALWORLD, MotionSense, UCI-HAR and SHL, ACESNet achieves accuracies of 95.37 ± 0.09%, 99.33 ± 0.12%, 98.01 ± 0.23% and 93.49 ± 0.10%, with Macro-F1 scores of 95.59 ± 0.09%, 99.12 ± 0.15%, 98.16 ± 0.21% and 94.03 ± 0.08%, respectively. A separate official subject-independent UCI-HAR evaluation yields 94.01 ± 0.84% accuracy and 94.11 ± 0.88% Macro-F1. The adaptive-kernel selector has a mean normalized entropy of 0.9114, providing evidence against severe single-branch collapse rather than strong kernel selectivity. On a Raspberry Pi 5 (Raspberry Pi Ltd., Cambridge, UK), ACESNet requires 8.731 MFLOPs and 1.606 ± 0.014 ms per window in FP32 ONNX Runtime 1.18.1. These results support a favorable performance–efficiency trade-off relative to the selected baselines under the stated protocol.

## 1. Introduction

Human activity recognition (HAR) is a core technology in ubiquitous computing and human–computer interaction that has demonstrated significant application potential in a wide range of domains, including healthcare monitoring (e.g., chronic disease management, rehabilitation assessment and fall detection), sports science (e.g., motion analysis and training optimization), intelligent environments (e.g., smart home control and environment-aware services), as well as virtual and augmented reality systems [1,2,3]. In recent years, two main paradigms—vision-based approaches [4] and sensor-based approaches [5]—have dominated HAR research. Vision-based HAR relies on images or videos captured by cameras to analyze human activities, but it inherently suffers from several limitations such as privacy concerns, sensitivity to lighting conditions and performance degradation due to viewpoint changes and occlusions [6]. With the rapid advancement of micro-electro-mechanical systems (MEMS) technology, inertial measurement units (IMUs), which typically integrate accelerometers, gyroscopes and magnetometers, have emerged as a promising alternative. Owing to their compact size, low power consumption and easy wearability (e.g., integration into wristbands, smartwatches, clothing, or specific body locations), IMUs can continuously provide raw motion and posture-related signals, such as tri-axial acceleration and angular velocity. These advantages make IMU-based sensing an ideal solution for non-intrusive, continuous and personalized activity recognition in real-world deployments.

However, accurately and robustly recognizing complex human activities directly from raw IMU signals remains highly challenging due to the multi-source, high-dimensional, noisy and highly variable nature of the data. Traditional SHAR approaches based on handcrafted feature engineering such as time-domain statistics and frequency-domain features [7] and combined with shallow machine learning classifiers, including support vector machines (SVMs) [8] and k-nearest neighbors (KNN) [9], have achieved reasonable performance in certain scenarios. Nevertheless, these methods rely heavily on manually designed features and domain expertise, leading to labor-intensive feature extraction pipelines and limited generalization capability. More importantly, shallow models struggle to effectively capture deep spatiotemporal dependencies and abstract motion patterns inherent in IMU data and often perform poorly when recognizing fine-grained activities (e.g., subtle gait variations and transitional movements) or previously unseen and undefined activities.

The emergence of deep learning (DL) techniques has provided strong momentum for overcoming these limitations. Owing to their end-to-end learning paradigm, DL models are capable of automatically learning highly abstract and discriminative feature representations directly from raw or minimally preprocessed IMU data, thereby effectively alleviating the reliance on handcrafted feature design. In particular, Recurrent Neural Networks (RNNs) and their variants, such as Long Short-Term Memory (LSTM) and Gated Recurrent Unit (GRU) networks [10,11], which are well suited for sequential data modeling, Convolutional Neural Networks (CNNs) that excel at capturing local spatiotemporal patterns, and hybrid architectures that combine the strengths of both (e.g., CNN-LSTM) [12], have demonstrated outstanding performance in processing IMU-based sensor data with strong temporal dependencies. These models are capable of modeling long-term motion dynamics and capturing contextual information of activities, thereby significantly improving recognition accuracy, robustness and generalization capability.

Consequently, IMU-based human activity recognition methods combined with deep learning models fully exploit the ubiquity and convenience of inertial sensors, together with the strong representation learning and pattern recognition capabilities of deep neural networks, thereby providing a solid technical foundation for high-accuracy, adaptive and scalable real-time activity sensing systems. Despite their impressive performance in SHAR tasks, deep neural networks are often associated with substantial computational overhead. In particular, recent studies have indicated that increasing model size is a key factor in improving recognition performance, which inevitably leads to a significant rise in computational cost [13,14]. Therefore, from the perspective of practical deployment and hardware constraints, it is essential to strike an appropriate balance between recognition accuracy and computational efficiency.

To address this challenge, several studies have attempted to balance performance and efficiency by designing lightweight convolutional architectures or efficient kernel functions. For example, ref. [15] introduced a multi-branch CNN kernel strategy to enhance representational capacity, while ref. [6] transitioned multi-branch convolutional modules to equivalent standard convolution operations to reduce inference-time overhead. In [16], attention mechanisms were incorporated to realize temporal–spatial dynamic convolution, enabling adaptive feature extraction across time and space. Moreover, ref. [17] introduced attention mechanisms into SHAR to capture inter-signal dependencies within time-series data. However, the quadratic computational complexity of self-attention results in considerable computational overhead. Although Split Depth-wise Transposed Attention (SDTA) [18] reduces complexity to some extent by modeling channel-wise covariance, its standard formulation still requires explicit computation of channel correlation matrices.

**Figure 1:**
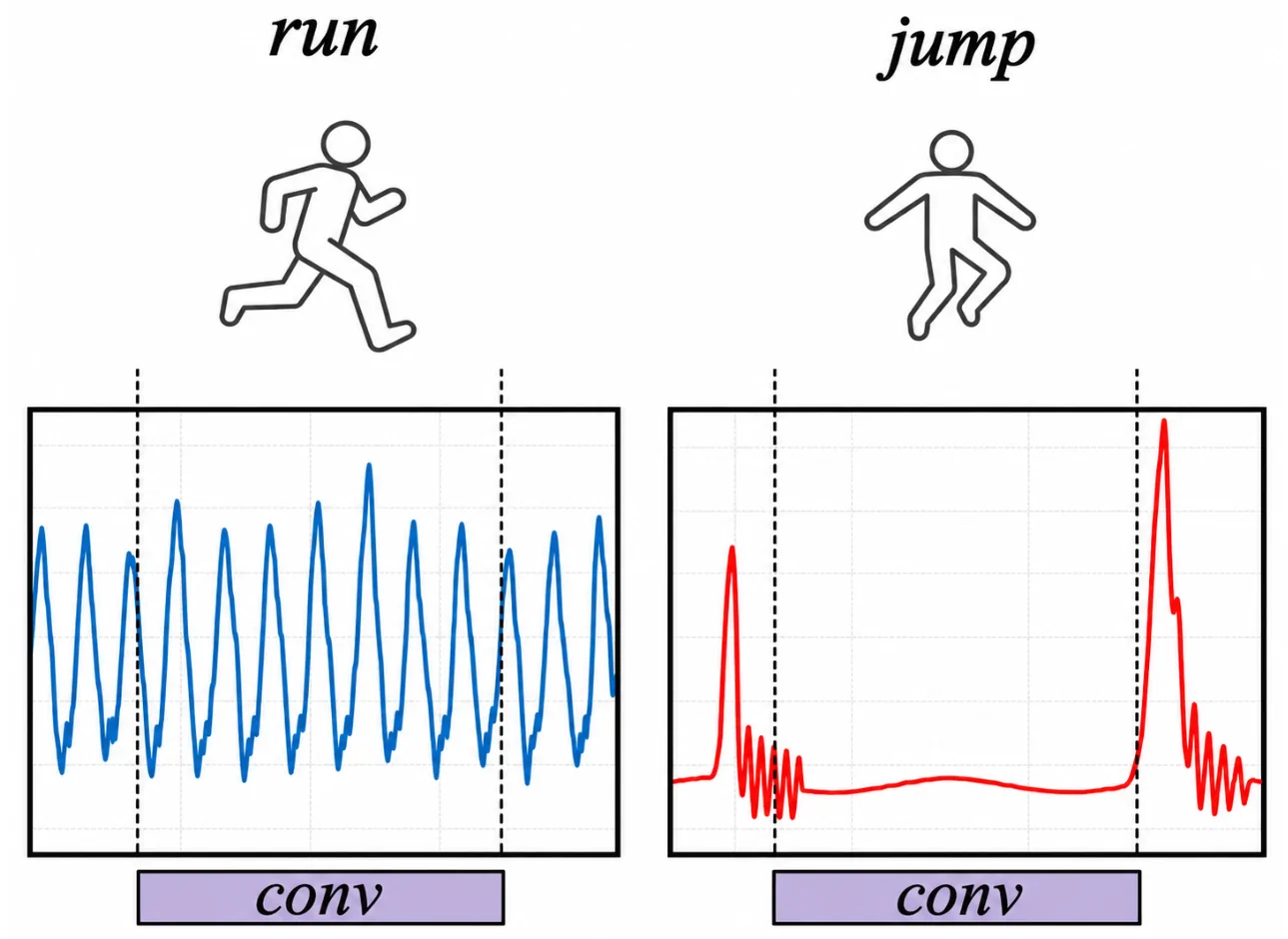
Single-kernel limitation. Blue and red curves schematically denote representative periodic running and transient jumping signal patterns, respectively.

The research gap addressed in this work is the joint problem of scale adaptation and deployment-aware temporal modeling. Wearable activities contain short transient patterns and longer periodic structures, whereas a conventional CNN applies a fixed temporal receptive field at each layer. Increasing depth or adding global attention can enlarge the receptive field, but it also increases computation and intermediate activation cost. The objective of ACESNet is therefore not to replace every recurrent or Transformer architecture, but to provide an adaptive convolutional alternative that can vary its local temporal scale while retaining a lightweight context-mixing path. As illustrated in Figure 1, a single kernel size can be suboptimal for activities with different temporal characteristics, such as periodic motion and short transient events. ACESNet addresses this issue through an Adaptive Kernel Encoder that dynamically combines several temporal convolution branches. Its Stage-3 design further combines multi-scale depthwise temporal mixing, explicit pointwise channel mixing and a parallel local CNN branch. Importantly, computational efficiency is evaluated separately from recognition accuracy using theoretical complexity, FLOPs, model size, peak memory and measured Raspberry Pi 5 inference latency.

The present study uses six inertial channels (three-axis acceleration and three-axis angular velocity). We therefore use the term “multi-channel inertial sensing” rather than the broader term “multimodal sensing” when referring to the ACESNet input. Multimodal fusion across heterogeneous sources can provide complementary information, as demonstrated in recent wearable systems combining distinct physiological and kinematic modalities [19]; however, such fusion also requires modality-wise validation to establish the contribution of each source. A systematic heterogeneous-modality study is outside the scope of the present six-axis IMU benchmark and is identified as future work.

We evaluate ACESNet on REALWORLD, MotionSense, UCI-HAR and SHL and compare it with the selected baseline implementations described in Section 4. The main contributions are summarized as follows:

1. We propose a three-stage architecture in which Adaptive Kernel Encoders dynamically combine kernels with k∈{3,5,7} in the first two stages. This provides input-dependent local temporal scale selection rather than a fixed single-kernel representation.
2. We develop a CNN-Enhanced Scale-fusion design that combines multi-scale depthwise temporal mixing with pointwise/grouped channel mixing, a parallel local CNN path and cross-scale feature recalibration. The design avoids explicitly forming a global T×T or C×C attention matrix; its practical efficiency is assessed by FLOPs and on-device latency rather than by parameter count alone.
3. We provide an evaluation protocol with a fixed data split, multiple random seeds, Macro-F1 and class-wise analysis, component-wise ablation, adaptive-kernel weight analysis, subject-independent verification and Raspberry Pi 5 deployment measurements. Performance claims are restricted to the selected baselines evaluated under the stated protocol.

The remainder of this paper is organized as follows. Section 2 reviews related work. Section 3 details the proposed ACESNet architecture. Section 4 presents the experimental setup, evaluation metrics, comprehensive performance analysis and ablation studies. Section 5 discusses the results. Finally, Section 6 concludes the paper and outlines future research directions.

## 2. Related Work

In this section, we review three lines of work most relevant to ACESNet: (1) wearable SHAR and edge-oriented models, (2) selective/dynamic convolution, and (3) attention and scale-fusion mechanisms.

**SHAR:** In recent years, deep learning has revolutionized SHAR by replacing manual feature engineering with end-to-end representation learning [20]. CNNs and RNNs have emerged as the dominant paradigms for processing wearable Inertial Measurement Unit (IMU) time-series data [21]. CNNs are widely utilized for their ability to extract local spatial features and scale-invariant patterns from sensor data. However, standard CNNs suffer from their fixed receptive field as a fundamental limitation. They struggle to simultaneously capture the short-term fluctuations in fine-grained activities (e.g., “jumping”) and the long-term dependencies of periodic motions (e.g., “walking”) without stacking excessive layers, which increases computational cost [22]. Recent works have explored multi-branch CNN architectures to capture features at different resolutions [23]. To address temporal dependencies, RNNs and their variants, such as Long Short-Term Memory (LSTM) and Gated Recurrent Units (GRUs) are often employed. Hybrid architectures such as CNN-LSTM [24] and CNN-BiGRU [25] have emerged as widely adopted benchmark models, in which CNNs extract spatial features, and RNNs capture the temporal evolution of sequential information. Xia et al. [26] proposed a CNN-LSTM framework that automatically learned temporal activity patterns from raw sensor streams. Their model achieved strong recognition performance across different datasets, demonstrating competitive robustness and efficiency. Gaud et al. [27] designed a multi-headed CNN-LSTM model for IMU-based gesture recognition and deployed a compressed version on Arduino Nano BLE, demonstrating real-time on-device inference for multiple activities under TinyML constraints. Despite their effectiveness, RNN-based models inherently lack parallelizability due to their sequential processing nature, leading to high latency on edge devices. Edge-oriented SHAR models address a related but distinct objective. For example, TinyHAR [28] reduces deployment cost through compact architectural design, while embedded demonstrations such as the compressed CNN–LSTM implementation of Gaud et al. [27] emphasize real-time feasibility. ACESNet differs in that its primary architectural mechanism is input-dependent temporal scale selection followed by convolutional scale fusion; the experiments therefore compare recognition accuracy and on-device cost separately instead of inferring deployment efficiency from model size alone.

**Dynamic Convolution Neural Networks:** To overcome the aforementioned limitations of fixed convolutional kernels and the inefficiency of RNNs, researchers have introduced dynamic convolution strategies and attention mechanisms into SHAR. Zhang et al. [29] integrated SE blocks into DeepConvLSTM to enhance the model’s sensitivity to critical sensor channels. While effective, these methods typically operate on static feature maps and do not alter the underlying convolution operation itself. A more recent advancement is Dynamic Convolution, which adapts the convolution kernels based on the input data. Li et al. [16] proposed the Temporal-Spatial Dynamic Convolution for SHAR, which dynamically learned kernel weights to adapt to the changing properties of sensor signals. Yin et al. [30] developed ConvBLSTM-PMwA, a parallel CNN-BiLSTM model equipped with an attention module, enabling robust feature extraction under noisy or incomplete sensor signals. Zou et al. [31] introduced STFNet, a spatiotemporal fusion framework that incrementally learned sensor features through staged extraction and local attention-based temporal modeling. Wang et al. [22] enhanced a CNN-based HAR framework with an attention mechanism that emphasized salient local patterns under weak supervision, achieving superior performance on weakly labeled datasets compared with DeepConvLSTM and standard CNN baselines. Adaptive Kernel Convolution has been explored to allow the kernel shape and sampling positions to deform to accommodate the irregular variations in time-series data, which significantly enhances feature extraction capability [32]. Technically, the Adaptive Kernel Encoder used here is closer to selective-kernel and dynamic-convolution families than to deformable convolution: several fixed candidate temporal kernels are evaluated in parallel and their outputs are combined by input-dependent Softmax weights. In contrast, CondConv [33] mixes expert parameters and Dynamic Convolution [34] aggregates convolution kernels themselves. ACESNet keeps the candidate operators explicit, which permits direct inspection of the k=3,5,7 selection weights at inference time (Section 4.4).

**Scale-fusion:** The Transformer architecture, underpinned by the self-attention mechanism, has shown exceptional performance in capturing global dependencies. In the SHAR domain, several Transformer variants have been proposed, such as MobileHARC [35] and TinyHAR [28], which attempt to balance global modeling with computational efficiency. However, standard multi-head self-attention tends to introduce considerable computational overhead as the sequence length increases, which becomes impractical for real-time SHAR deployment on wearable devices. Motivated by the need for efficient attention designs, Maaz et al. [18] introduced EdgeNeXt, a hybrid architecture featuring the Split Depth-wise Transpose Attention (SDTA) encoder, which forms attention in the embedded channel dimension rather than the token dimension. This changes the dominant attention term from $O(T^{2}C)$ to $O(C^{2}T)$ but does not imply that one term is universally smaller: the relative cost depends on the embedded channel width *C* and temporal length *T* at the stage where the operator is applied. ACESNet therefore does not assume that channel covariance is the unique bottleneck in six-axis SHAR. Instead, its CNN-Enhanced Scale-fusion design uses convolutional context mixing that scales linearly with temporal length and avoids materializing a global attention matrix. The formal comparison and measured FLOPs/latency are provided in Section 3.4.

## 3. Methodology

**Figure 2:**
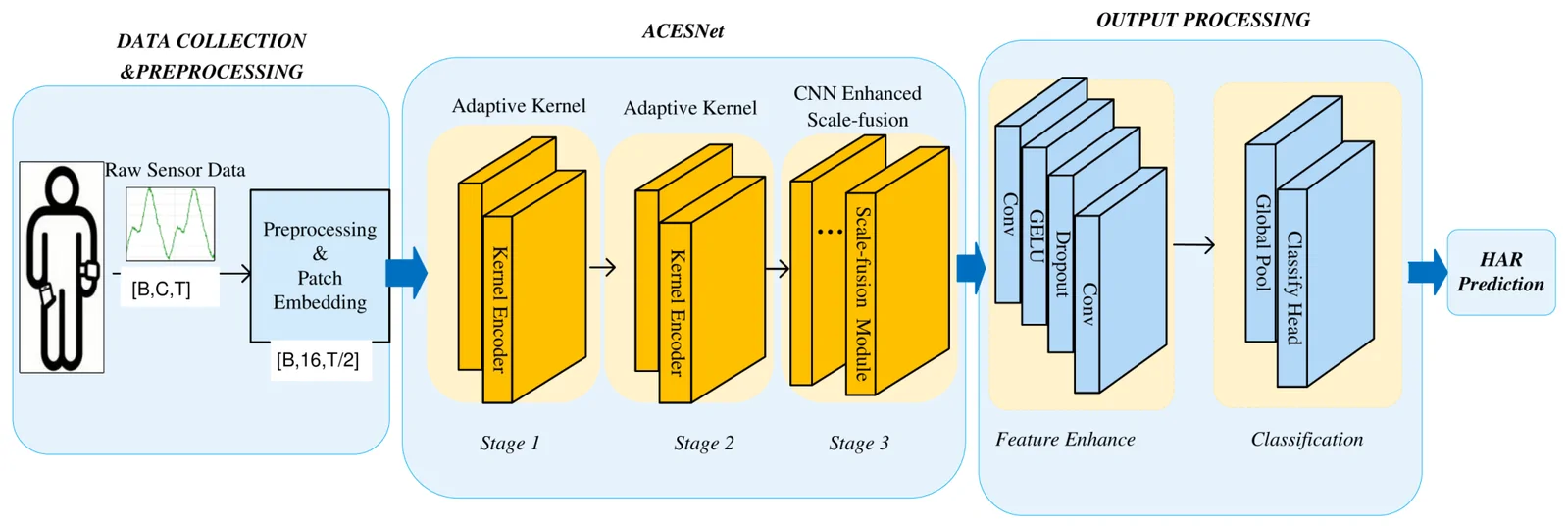
ACESNet architecture. Ellipses denote repeated identical blocks rather than omitted unique operations.

In this section, ACESNet is introduced for the SHAR task. As illustrated in Figure 2, the proposed network adopts a three-stage progressive feature abstraction strategy. The raw input signals are generated by sensors embedded in smart devices, from which discriminative feature representations are extracted and subsequently fed into a classification network for activity recognition. Specifically, the fixed-length inertial input windows are mapped into a high-dimensional feature space through a patch embedding module. The resulting features are subsequently processed by ACESNet, where the channel dimensionality is progressively increased while sufficient temporal resolution is preserved, enabling effective information reconstruction across stages for human activity recognition. The overall architecture can be mathematically formulated as follows, where $X^{\left(i\right)}$ represents different stages of our network and $X^{\left(0\right)}$ denotes the initial feature representation after patch embedding.

(1) $$X^{\left(i\right)}={Stage}_{i}(X^{\left(i-1\right)})\;\in R^{B\times C_{i}\times T_{i}},i\in \left\{1,2,3\right\}$$

The dimensional transformations at each stage are detailed in Table 1. The architecture is fully convolutional along the temporal axis. Hence, *T* denotes the temporal length of the fixed-length input tensor supplied to the network rather than a hard-coded architectural requirement; the temporal dimension is reduced by a factor of two at patch embedding and at each subsequent stage transition. In the experiments reported here, all four input tensors contain 128 samples per window, and the common runner does not construct additional windows after loading.

For the reported ACESNet configuration, Stage 3 first applies a transition consisting of LayerNorm followed by a k=2, stride-2 Conv1D that maps 32→64 channels, BatchNorm and GELU, thereby converting 32×T/4 to 64×T/8. This transition is followed, in order, by six identical CNN-Enhanced Scale-fusion blocks; all convolutions inside these six blocks use a stride of 1 and therefore preserve the 64×T/8 resolution. Within each Stage-3 block, the Scale-fusion path uses five depthwise temporal kernels k∈{1,3,5,7,9} with g=C=64 groups. The parallel CNN path uses grouped temporal convolutions with k∈{3,5}, each mapping 64→32 channels with g=16=C/4, followed by BatchNorm, GELU and a pointwise 32→64 projection. The cross-scale gate uses pointwise projections 64→16→64, and the post-fusion MLP uses 64→224→64 (expansion ratio 3.5). No additional downsampling occurs within the six Stage-3 blocks.

The remainder of this section is organized as follows. Section 3.2 presents the proposed Adaptive Kernel Encoder. We first motivate the design of the Adaptive Kernel Encoder through an analysis of the characteristics of the SHAR task and then describe the Adaptive Kernel Encoder layer, which consists of an adaptive multi-scale convolutional kernel fusion module and an optimization module based on SE-style channel attention. Section 3.3 introduces the CNN-Enhanced Scale-fusion Module. At this stage, a Multi-Scale Convolutional Fusion strategy is employed to compensate for the limitations of pure temporal modeling in capturing fine-grained local patterns, while preserving the network’s advantages in temporal modeling and long-range dependency capture. Through the deep integration of two complementary feature extraction paradigms, the proposed framework enables semantic-level feature representation learning.

### 3.1. Effective Temporal Receptive Field

The kernel width of one isolated layer is not the effective receptive field of a stacked, downsampled network. Let $R_{l}$ denote the cumulative receptive field (in raw input samples) before layer *l*, and let $j_{l}$ denote the effective sampling jump. For a temporal convolution with kernel width $k_{l}$ and stride $s_{l}$,

(2) $$R_{l+1}=R_{l}+\left(k_{l}-1\right)j_{l},\;j_{l+1}=j_{l}s_{l}.$$

Using the largest temporal branch (k=7) in the first two stages, the 7-tap stride-2 patch embedding gives a cumulative receptive field of 7 samples; after the two Stage-1 encoders, the receptive field is 31 samples, and after the two Stage-2 encoders, it is 79 samples. At 50 Hz, 79 samples correspond to 1.58 s. Accordingly, Stage 2 performs mid-range temporal aggregation, with a cumulative receptive field of 79 samples (1.58 s at 50 Hz) provided by the stacked, downsampled architecture rather than by any single convolutional layer. Stage 3 further expands the theoretical receptive field beyond the 128-sample input window, so its effective support is capped by the available window. The cumulative receptive fields are summarized in Table 2.

### 3.2. Adaptive Kernel Encoder

#### 3.2.1. Motivation

For raw IMU signals containing high-frequency transients and activities characterized by diverse temporal and motion scales, convolution kernels with fixed receptive fields are insufficient to simultaneously accommodate the feature extraction requirements of different activities. To address this limitation, we employ an Adaptive Kernel Encoder that dynamically selects appropriate receptive fields via a learnable attention mechanism [6]. Inspired by [33,34], an operator space composed of convolutional kernels at three different scales is constructed to cover the diverse frequency characteristics of human activities. Given an input feature $x\in R^{B\times C\times T}$, the multi-kernel convolution operation is defined accordingly:

(3) $$y_{k}=\;Conv1D_{k}\left(x\right),k\in \left\{3,5,7\right\}$$

where $Conv1D_{k}$ denotes a one-dimensional convolution operation with kernel size *k*. The resulting feature maps $y_{k}$ capture local patterns at different temporal scales, enabling the model to adaptively extract features from activities with varying frequency characteristics.

#### 3.2.2. Adaptive Multi-Scale Convolutional Kernel Fusion

To achieve effective fusion of features across different temporal scales, an attention mechanism is introduced to learn the global statistical properties of the input signal and dynamically generate the weights associated with each convolutional kernel. Specifically, Global adaptive Average Pooling (GAP) is applied to extract global contextual information from the input features, compressing the temporal dimension into a channel-wise descriptor *g*:

(4) $$\;g=GAP\left(x\right)=\frac{1}{T}\sum_{t=1}^{T}x\left[:,:,t\right]$$

A lightweight linear projection then maps *g* into the kernel-weight space. Let $W_{k}\in R^{C\times C}$ denote the learnable weight matrix, and the kernel scores $S=\omega_{k}^{T}\cdot g$ are obtained via an inner product between the projected features and the kernel weights. To ensure that the resulting weights form a valid probability distribution, a Softmax function is applied to obtain the final attention weights $a_{k}$. The output of the encoder *y* is computed as a weighted linear combination of the feature maps from different scales:

(5) $$\begin{array}{cc} a_{k} & =\frac{\exp(S_{k})}{\sum_{j\in \{3,5,7\}}\exp\left(S_{j}\right)},\;k\in \left\{3,5,7\right\},\\ y & =\sum_{k\in \{3,5,7\}}a_{k}\;y_{k}. \end{array}$$

This dynamic fusion mechanism integrates the candidate temporal scales using input-dependent weights. Because a standard Softmax can in principle become highly concentrated, the evaluation explicitly measures the test-set distribution and normalized entropy of $a_{k}$ rather than assuming that all branches remain active (Section 4.4).

#### 3.2.3. SE-Style Channel Attention

**Figure 3:**
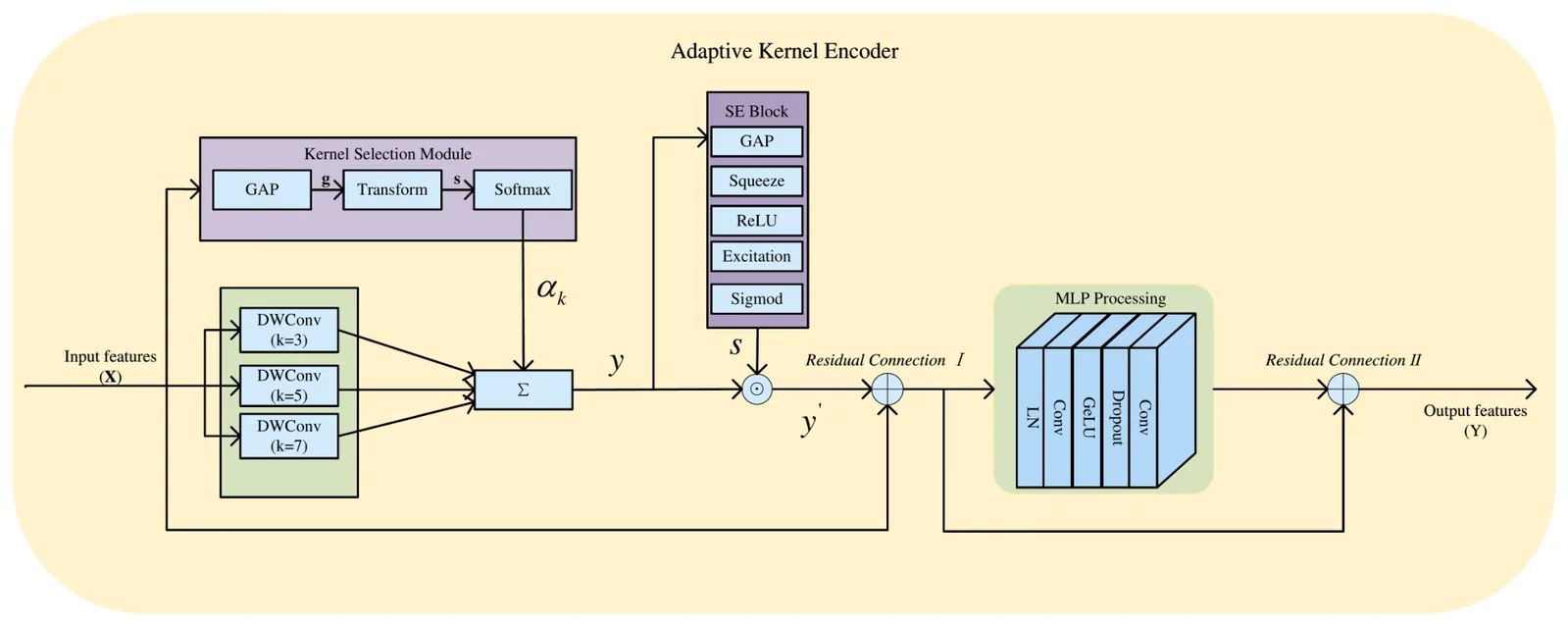
Structure of the Adaptive Kernel Encoder.

Following multi-kernel feature fusion, a Squeeze-and-Excitation (SE) module [16,36] is further incorporated to enhance inter-channel dependency modeling. The SE attention mechanism is formulated accordingly:

(6) $$\begin{array}{cc} s & =\sigma \;\left(W_{2}\;ReLU\;\left(W_{1}\;GAP\left(y\right)\right)\right),\\ y^{\prime } & =s⊙y. \end{array}$$

where σ denotes the sigmoid activation function, $W_{1}\in R^{\frac{C}{r}\times C}$ and $W_{2}\in R^{C\times \frac{C}{r}}$ represent the weights of the squeeze and excitation layers, and *r* is the reduction ratio used to decrease dimensionality, limit model complexity and improve generalization. The symbol ⊙ denotes element-wise multiplication. To ensure training stability in deep architectures and alleviate gradient vanishing, the entire encoder module is wrapped within two cascaded residual connections, which facilitate lossless information propagation and preserve the original feature representations. The overall architecture of the Adaptive Kernel Encoder is illustrated in Figure 3.

### 3.3. CNN-Enhanced Scale-Fusion Module

**Figure 4:**
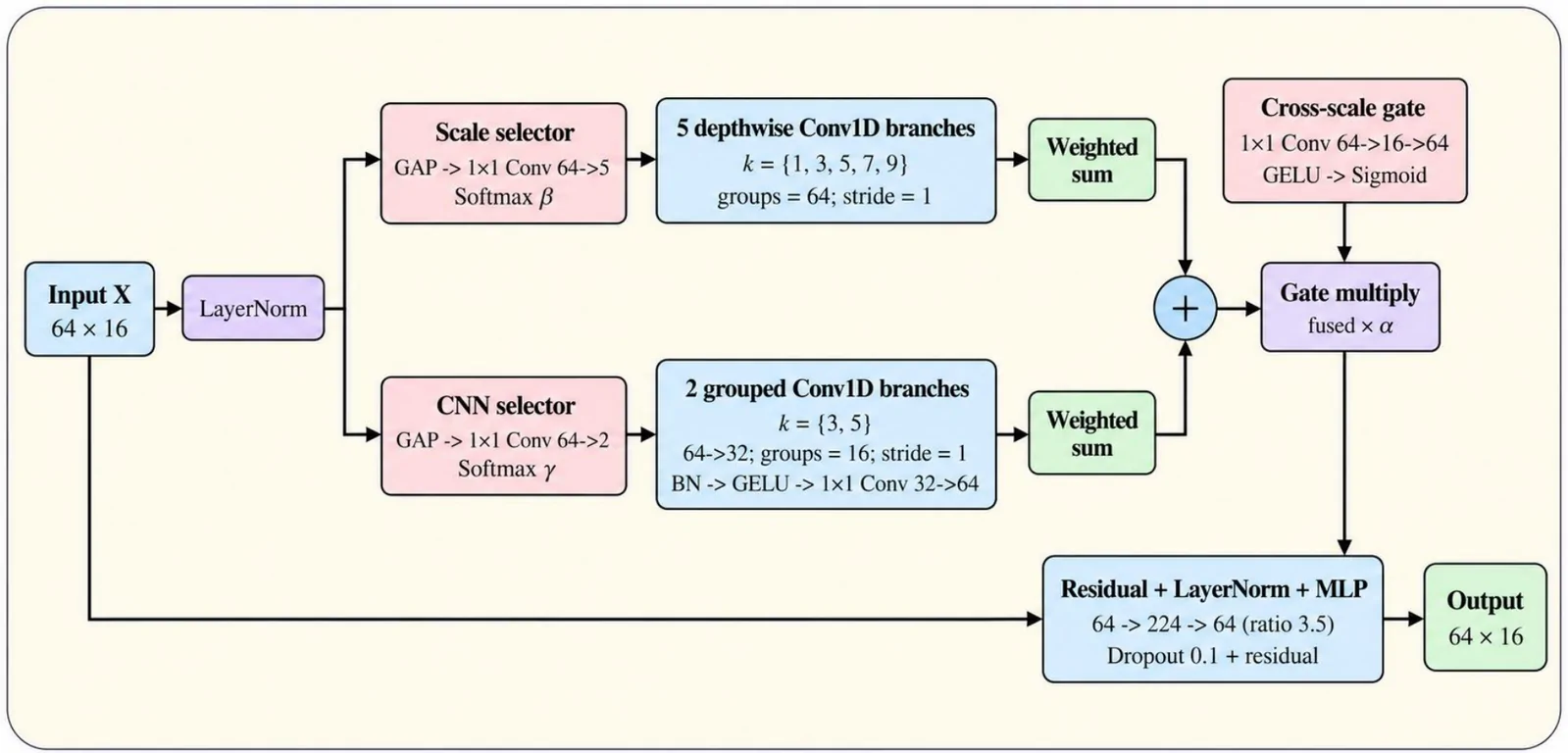
Implemented CNN-Enhanced Scale-fusion block used in Stage 3.

The third stage is responsible for abstracting features from the preceding stages into high-level semantic representations. To address the limitations of purely Transformer-based architectures in capturing fine-grained local patterns, as well as the shortcomings of CNNs in global context modeling, we propose a CNN-Enhanced Scale-fusion module. This module introduces a parallel CNN branch within the Scale-fusion architecture to achieve complementary feature learning. Specifically, the Scale-fusion branch applies multi-scale depthwise temporal convolutions and Softmax scale selection, while the parallel CNN branch uses grouped temporal convolutions followed by pointwise (1×1) convolutions to restore explicit channel mixing. The two streams are fused and recalibrated by a lightweight cross-scale gate. The temporal context-mixing terms scale linearly with *T*; the full block also contains pointwise channel projections whose cost depends on $C^{2}$, as quantified in Section 3.4. The overall architecture is illustrated in Figure 4.

The SDTA mechanism was originally developed for two-dimensional computer vision tasks [18]. To make the tensor convention explicit, let $Q,K,V\in R^{B\times C\times T}$ and let $K^{⊤}\in R^{B\times T\times C}$ denote transposition of the last two dimensions. Under this convention, channel-wise attention is

(7) $$A=Softmax\;\left(\frac{QK^{⊤}}{\sqrt{T}}\right)\in R^{B\times C\times C},\;Y=AV\in R^{B\times C\times T}.$$

Thus, $QK^{⊤}$, rather than $K^{⊤}Q$, forms the C×C channel-attention matrix by aggregating over the temporal dimension. This notation is used only to describe the reference transposed-attention operation; the implemented ACESNet Stage-3 block replaces the explicit global attention matrix with the convolutional Scale-fusion mechanism described below. However, explicitly computing global attention not only incurs a high computational cost, but also increases the risk of overfitting to spurious and incidental inter-sensor correlations. To mitigate these issues, we propose a Multi-Scale Convolutional Fusion scheme that replaces explicit global attention with convolution-based multi-scale fusion. This design encourages the model to focus on local temporal context, which better conforms to the physical characteristics of human motion signals. Specifically, a multi-scale spatial mixing strategy is adopted, in which the input features are processed by depthwise convolutional kernels with different receptive field sizes k∈{1,3,5,7,9}. This enables the model to simultaneously capture point-wise characteristics, short-term fluctuations and long-term temporal trends. Subsequently, dynamic channel-wise feature reweighting is performed without explicitly computing an attention map, thereby reducing the computational complexity:

(8) $$\begin{array}{cc} Y_{s,k} & =DWConv_{k\times 1}\left(X_{in}\right),\;k\in \left\{1,3,5,7,9\right\},\\ \beta & =Softmax\;\left(W_{\beta }\;GAP\left(X_{in}\right)\right),\\ Z_{S-f} & =\sum_{k\in \{1,3,5,7,9\}}\beta_{k}\;Y_{s,k}. \end{array}$$

Here, $DWConv_{k\times 1}$ denotes a one-dimensional depthwise convolution of width *k*. The Softmax weights $\beta_{k}$ are generated from the pooled input and sum to one across the five temporal scales.

A parallel CNN branch is introduced to focus on high-frequency details. This branch employs group convolution (GC), batch normalization (BN) and the GELU activation function to capture local signal variations and abrupt changes, while simultaneously computing convolutional kernel attention to ensure that critical transient information is preserved and not smoothed out by the Scale-fusion branch:

(9) $$\begin{array}{cc} Y_{c,k} & =PWConv_{1\times 1}\;\left(GELU\;\left(BN\;\left(GC_{k\times 1}\left(X_{in}\right)\right)\right)\right),\;k\in \left\{3,5\right\},\\ \gamma & =Softmax\;\left(W_{\gamma }\;GAP\left(X_{in}\right)\right),\\ Z_{CNN} & =\sum_{k\in \{3,5\}}\gamma_{k}\;Y_{c,k}. \end{array}$$

Here, $GC_{k\times 1}$ denotes grouped temporal convolution, and $PWConv_{1\times 1}$ denotes pointwise channel mixing. In Stage 3, where C=64, each grouped branch maps 64→32 channels using g=16=C/4 groups and stride 1, followed by the pointwise 32→64 projection. The two CNN branches use k=3 and k=5, consistent with Figure 4.

To effectively integrate information from the two branches, a cross-scale attention enhancement mechanism is adopted to achieve complementary feature fusion. Specifically, an initial linear fusion $Z_{fused}$ is first applied, followed by a lightweight attention network inspired by the SE module to learn the relative importance distribution within the fused features, yielding the final output:

(10) $$\begin{array}{cc} Z_{fused} & =Z_{S-f}+Z_{CNN},\\ a & =\sigma \;\left(W_{b}\;GELU\;\left(W_{a}Z_{fused}\right)\right),\\ Z_{enhanced} & =a⊙Z_{fused} \end{array}$$

For the implemented Stage-3 width C=64, the cross-scale gate uses pointwise projections 64→16→64 with GELU and sigmoid activation. The enhanced feature is added residually to the block input and then refined by a second LayerNorm and a pointwise MLP 64→224→64 with GELU and Dropout 0.1, followed by the second residual connection. Stage 3 therefore preserves the 64×16 feature shape for a 128-sample input. In summary, the proposed module combines multi-scale temporal filtering with explicit channel mixing and local feature extraction. The present study does not claim experimentally demonstrated robustness to sensor noise because controlled sensor corruption is not isolated experimentally. Such robustness is therefore listed as a limitation and future validation direction in Section 5.

### 3.4. Computational Complexity

To avoid conflating parameter count with computational efficiency, we compare the context-mixing operations using a common input $X\in R^{B\times C\times T}$. Token self-attention forms a T×T affinity matrix and has an attention-product term of $O(BT^{2}C)$, whereas transposed/channel attention forms a C×C matrix with term $O(BTC^{2})$. These expressions refer to the affinity/mixing operation; the linear Q/K/V projections add $O(BTC^{2})$ terms in both cases.

For the implemented CNN-enhanced block, let $K_{s}=\left\{1,3,5,7,9\right\}$ denote the depthwise Scale-fusion kernels and $K_{c}=\left\{3,5\right\}$ the parallel CNN kernels. The depthwise Scale-fusion cost is

(11) $$C_{SF}=BTC\sum_{k\in K_{s}}k.$$

In each parallel CNN branch, the grouped convolution maps *C* channels to C/2 channels using g=C/4 groups, followed by a pointwise C/2→C projection. Therefore,

(12) $$C_{CNN}=BT\sum_{k\in K_{c}}\left(2kC+\frac{C^{2}}{2}\right).$$

The cross-scale gate uses C→C/4→C pointwise projections, giving $C_{gate}=\frac{1}{2}BTC^{2}$. The post-fusion MLP uses an expansion ratio of 3.5, giving $C_{MLP}=7BTC^{2}$. The Softmax selectors operate on globally pooled descriptors and contribute only lower-order O(BC|K|) terms. Thus, the implemented block is linear in temporal length *T* but retains pointwise channel-mixing terms proportional to $C^{2}$. We therefore do not describe the full block as linear in both *T* and *C*, nor do we identify channel covariance as a universal bottleneck for six-axis SHAR. Table 3 summarizes the principal context-mixing terms.

At Stage 3 of ACESNet with a 128-sample input, the embedded feature shape is approximately C=64 and T=16. We emphasize that C=64 is the learned feature width, not the six raw sensor axes. Because theoretical operation counts do not capture hardware-specific efficiency of depthwise/grouped convolution versus attention kernels, the experiments additionally report measured FLOPs, model size, memory and Raspberry Pi 5 latency later in the experimental evaluation.

## 4. Experiments

**Figure 5:**
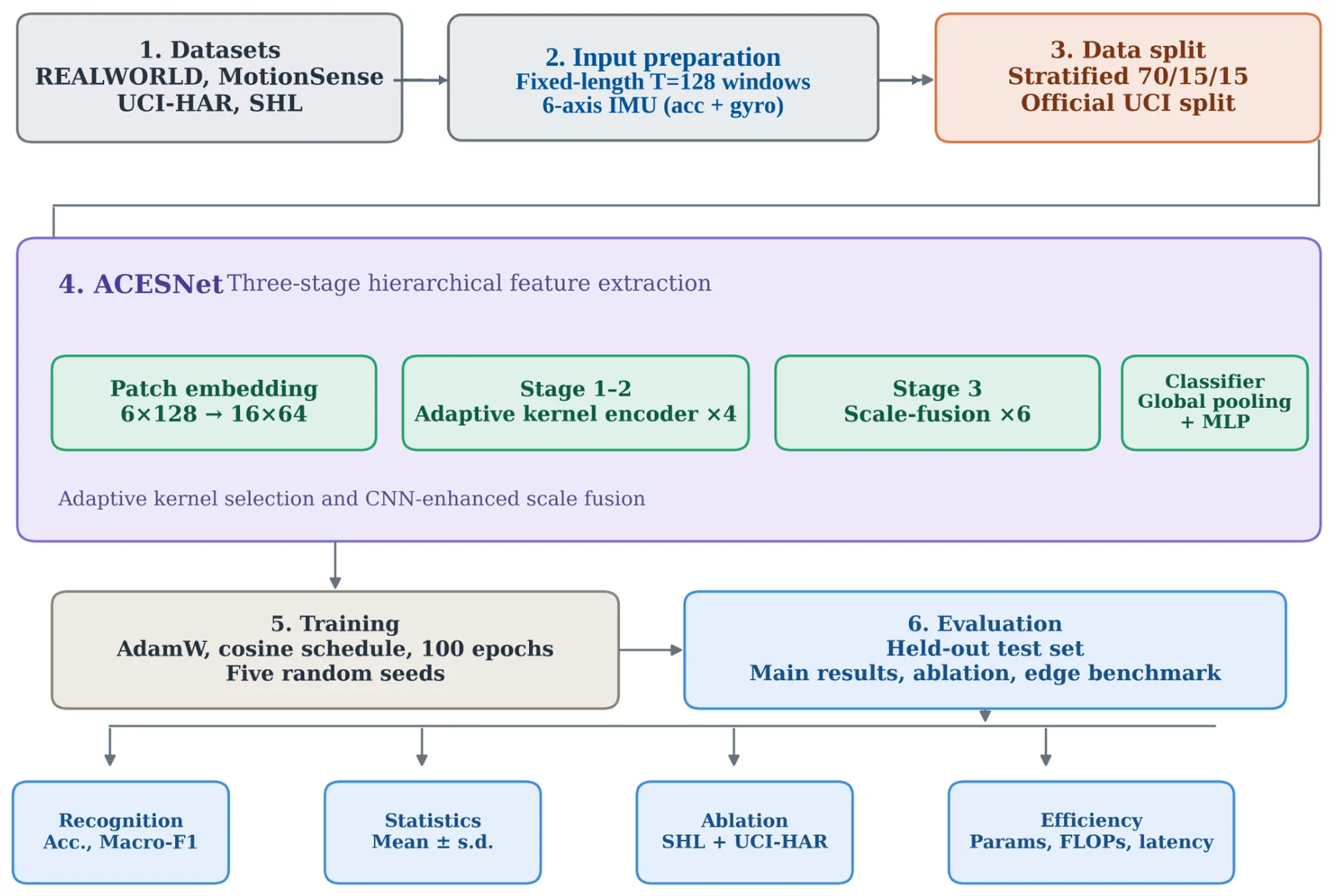
Experimental workflow from fixed-length input tensors to model evaluation.

In this section, ACESNet is evaluated on four public SHAR datasets using a common data-splitting and metric pipeline. Figure 5 summarizes the evaluation protocol from prepared sensor windows through model evaluation and edge-device benchmarking. Comparative claims are restricted to the selected baselines evaluated in this study.

### 4.1. Experimental Setup

#### 4.1.1. Datasets

Experiments were conducted on four public SHAR datasets: REALWORLD, MotionSense, UCI-HAR and SHL. The experimental pipeline began from fixed-length inertial input tensors containing six channels (three accelerometer axes and three gyroscope axes) and T=128 samples per window. After loading, the common runner only converted the arrays to channel-first format [N,C,T], retained the six inertial channels and applied the fixed train/validation/test split. It did not construct additional windows or apply post-load resampling, filtering, missing-value imputation or normalization. Consequently, no additional window stride or overlap was introduced by the present implementation, and no normalization statistics were estimated from the training, validation or test subsets by the common runner. Sensor-location metadata were not used as an input or preprocessing variable by the runner, and no location-specific remapping was performed after loading. All compared models therefore received exactly the same numerical tensors and split indices.

REALWORLD [37] contains recordings from 15 participants performing eight activities under real-world conditions. The original resource provides multiple sensor types and body locations. In the present benchmark, only the six accelerometer–gyroscope channels retained in the prepared input file were used. Although the source signals were sampled at 50 Hz, the prepared benchmark tensor used in this study contained 128 samples per window.

MotionSense [38] contains smartphone motion data from 24 participants performing six activities. The present experiments used the accelerometer and gyroscope channels represented by 128-sample input windows. UCI-HAR [39] contains 30 participants and six activities recorded at 50 Hz; the same six inertial channels and 128-sample windows were used. SHL [40] contains data from three participants and eight locomotion/transportation classes (Still, Walking, Run, Bike, Car, Bus, Train and Subway); the benchmark used the six selected inertial channels and 128-sample windows.

For the main comparison, the prepared windows of each dataset were partitioned by a fixed stratified *window-level* split into 70% training, 15% validation and 15% testing subsets using split seed 42. The split seed was held fixed across all models and training seeds, which guaranteed identical test samples for paired model comparisons. Because a window-level split does not by itself guarantee subject exclusivity, Section 4.5 separately reports subject-independent verification using the official UCI-HAR subject split. The two protocols are reported separately throughout the analysis. Table 4 summarizes the dataset inputs and post-load processing used by the common runner.

#### 4.1.2. Evaluation Metrics

Because SHL and several class distributions were imbalanced, the evaluation did not rely on overall accuracy alone. The primary recognition metrics were Accuracy and Macro-F1, with Macro-F1 assigning equal weight to each activity class. The five-seed row-normalized confusion matrices additionally provide class-wise recall patterns, and Balanced Accuracy is reported for the subject-independent UCI-HAR verification. For a *K*-class problem,

(13) $$\begin{array}{cc} Accuracy & =\frac{\sum_{k=1}^{K}TP_{k}}{N},\\ F1_{k} & =\frac{2\;Precision_{k}\;Recall_{k}}{Precision_{k}+Recall_{k}},\\ MacroF1 & =\frac{1}{K}\sum_{k=1}^{K}F1_{k}. \end{array}$$

All repeated-run recognition results are reported as mean ± sample standard deviation across independent training seeds. Computational efficiency was evaluated independently using parameter count, FLOPs, serialized model size, memory and inference latency/throughput on a Raspberry Pi 5 (Raspberry Pi Ltd., Cambridge, UK). Five-seed row-normalized confusion matrices were used to inspect class-specific errors.

#### 4.1.3. Experimental Details

The principal comparison included five selected baseline architectures (DanHAR, C3CNN, DBC, TASKED and TCN-Attention) together with ACESNet. All six methods used the same prepared six-channel windows, the same fixed stratified 70/15/15 split indices (split seed 42), the same 100-epoch maximum training budget and the same five training seeds (0–4). The training seed changed initialization and stochastic optimization but never changed the data partition.

ACESNet, C3CNN and TCN-Attention were trained in PyTorch 2.8.0 (CUDA 12.8 build) on Ubuntu 22.04.5 using an Intel^®^ Xeon^®^ Silver 4410Y CPU (Intel Corporation, Santa Clara, CA, USA), 128 GB RAM and an NVIDIA RTX 4090 GPU (NVIDIA Corporation, Santa Clara, CA, USA). Their common runner used AdamW, a batch size of 512, an initial learning rate of $10^{-3}$, weight decay $10^{-4}$, five warm-up epochs, cosine decay, gradient clipping at 1.0 and label smoothing of 0.1; mixed precision was enabled on the GPU. DanHAR, DBC and TASKED retained their TensorFlow 2.15.1/Keras 2.15.0 implementations and were trained with Adam at $10^{-3}$, a batch size of 64, early stopping on validation accuracy and ReduceLROnPlateau. This preserved architecture-specific implementations while controlling the data, split, epoch budget, seed set and evaluation metrics. Table 5 summarizes the settings that differ across the two implementation groups.

### 4.2. Comparative Experiments

Table 6 reports the five-seed comparison under the fixed window-level split. ACESNet has the highest mean Accuracy and Macro-F1 on all four datasets, although the margins on the easiest benchmark and on SHL are small. Accordingly, the results are interpreted as performance under the stated protocol rather than as universal state-of-the-art evidence.

#### 4.2.1. Results on REALWORLD and MotionSense

On REALWORLD, ACESNet reaches 95.37 ± 0.09% Accuracy and 95.59 ± 0.09% Macro-F1. The strongest selected baseline is TCN-Attention, with 95.08 ± 0.14% Accuracy and 95.49 ± 0.13% Macro-F1; the corresponding mean margins are 0.29 and 0.10 percentage points. On MotionSense, ACESNet obtains 99.33 ± 0.12% Accuracy and 99.12 ± 0.15% Macro-F1. DanHAR has the next-highest mean Accuracy (99.11%), whereas TCN-Attention has the next-highest Macro-F1 (99.02%). The narrow margins on MotionSense indicate that the dataset is close to saturation for several of the evaluated models.

#### 4.2.2. Results on UCI-HAR and SHL

On UCI-HAR, ACESNet achieves 98.01 ± 0.23% Accuracy and 98.16 ± 0.21% Macro-F1, compared with 97.62 ± 0.29% and 97.81 ± 0.27% for DBC, the strongest baseline by mean UCI-HAR Accuracy and Macro-F1. On SHL, ACESNet reaches 93.49 ± 0.10% Accuracy and 94.03 ± 0.08% Macro-F1. C3CNN is very close at 93.45 ± 0.16% and 93.94 ± 0.15%, so the SHL result should be viewed as a small but consistent mean improvement rather than a large separation. The class-wise behavior on SHL is examined further using the five-seed normalized confusion matrix in Section 5.

### 4.3. Ablation Studies

To isolate the principal configurable components, the ablation was performed on SHL and UCI-HAR using three training seeds (0–2) and the same split seed 42. The study compared a conventional CNN, a fixed-kernel ACESNet control, removal of the kernel-encoder channel attention, removal of the parallel CNN branch and the full model. The fixed-kernel control retained almost the same model capacity as the full network (0.2609M versus 0.2623M parameters), which reduced the capacity confound when assessing kernel adaptivity. Cross-scale recalibration was retained as part of the Scale-fusion implementation and was not assigned an independent quantitative contribution in this study.

On SHL, removing the parallel CNN branch produced the largest Macro-F1 reduction relative to the full model (94.02 to 93.29), followed by replacing adaptive kernels with fixed kernels (94.02 to 93.48) and removing kernel channel attention (94.02 to 93.74). On UCI-HAR, the differences were smaller: the full model reached 98.12 Macro-F1, while the fixed-kernel, no-channel-attention and no-parallel-CNN variants obtained 97.81, 98.00 and 97.76, respectively. The fact that the 0.2609M fixed-kernel control did not match the full model on either dataset indicates that the gain cannot be explained solely by adding parameters.

### 4.4. Adaptive Kernel Weight Analysis

**Figure 6:**
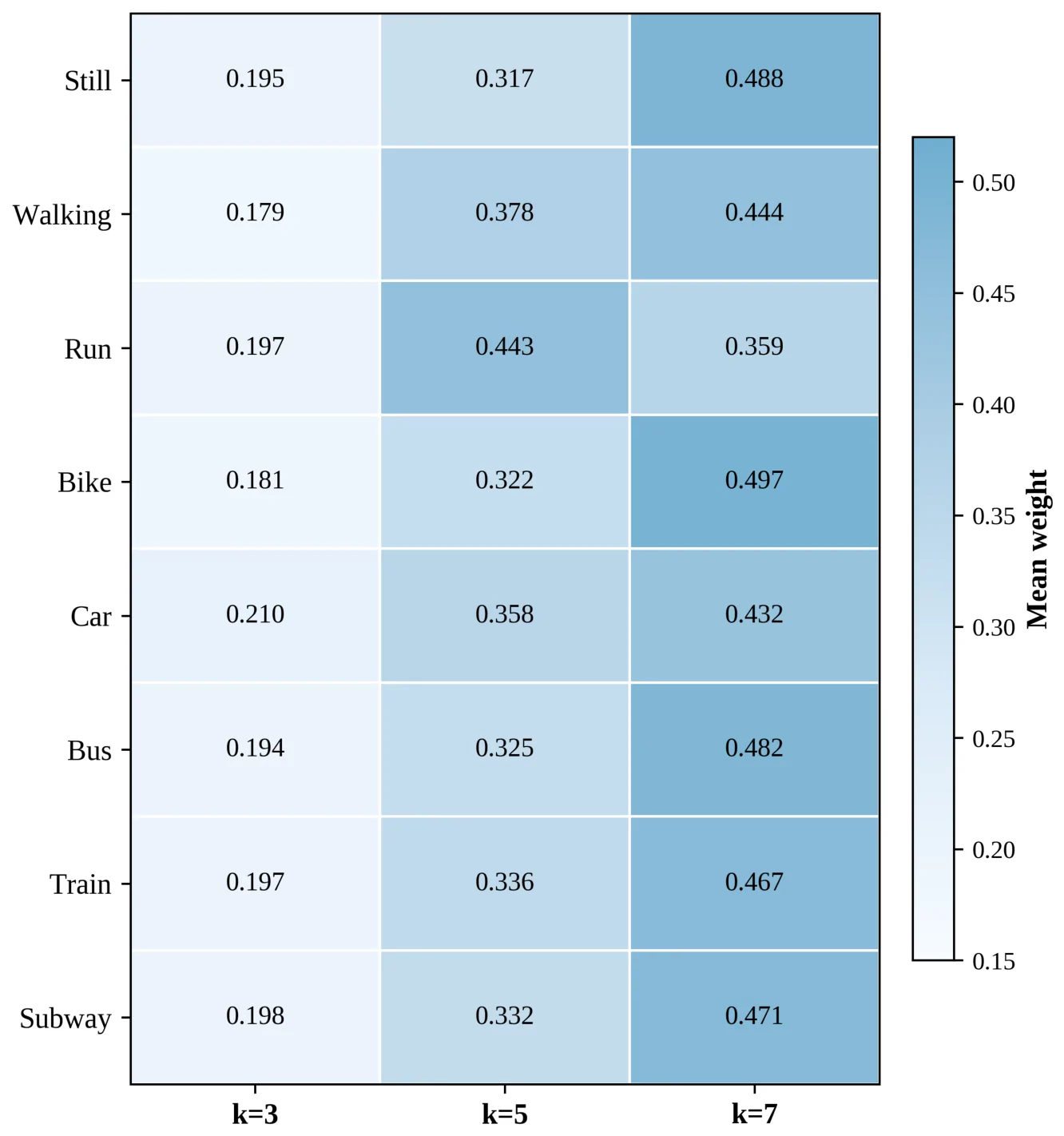
Adaptive-kernel weights on SHL.

The adaptive kernel weights were recorded during inference without retraining. For every test window, we collected $a_{3}$, $a_{5}$ and $a_{7}$ from the Adaptive Kernel Encoder and aggregated them by activity class. We also report the normalized entropy

(14) $$H_{norm}=-\frac{1}{\log3}\sum_{k\in \{3,5,7\}}a_{k}\log\left(a_{k}+\epsilon \right),$$

where $H_{norm}=0$ corresponds to complete single-branch collapse and $H_{norm}=1$ to a uniform three-branch distribution. The mean normalized entropy over the monitored encoder layers was 0.9114. Because this value was relatively close to the uniform-distribution limit, its primary interpretation was that all three candidate branches remained jointly utilized and that severe single-branch collapse was not observed; it should not be interpreted as evidence of strong or sharply selective kernel routing. The class-averaged weights nevertheless showed modest activity-dependent preference shifts. For example, Run placed relatively more weight on k=5 (0.44), whereas k=7 received the largest average weight for Still (0.49), Bike (0.50), Bus (0.48), Train (0.47) and Subway (0.47). Figure 6 visualizes these modest class-dependent differences.

### 4.5. Subject-Independent Verification

The main comparison used a fixed stratified window-level split for reproducibility and paired model comparison. Because this protocol can place windows from the same participant in different subsets, we additionally evaluated ACESNet using the official subject-disjoint UCI-HAR training/test partition. This verification is reported separately rather than describing the main benchmark as subject-independent.

The subject-independent Accuracy was lower than the 98.01% mean obtained with the window-level benchmark, as expected when participant overlap was removed. Nevertheless, the result remained above 94% for both Accuracy and Macro-F1, showing that ACESNet retained substantial cross-subject recognition capability while also quantifying the optimism introduced by the easier window-level protocol.

### 4.6. Computational and Edge-Device Efficiency

The efficiency analysis separated arithmetic complexity from measured runtime behavior. All six models were exported to ONNX and executed with the same ONNX Runtime 1.18.1 CPU backend on the Raspberry Pi 5 in FP32, batch size of one and one CPU thread. After 100 warm-up iterations, 1000 inference runs were timed for each model. FLOPs use the convention that one multiply–accumulate (MAC) corresponds to two floating-point operations, and Peak RSS denotes the maximum resident process memory observed during the benchmark.

ACESNet had the lowest parameter count among the six exported models and the second-lowest measured latency, behind TASKED. Its 8.731 MFLOPs were only modestly above C3CNN (6.783 MFLOPs) and substantially below TASKED, TCN-Attention, DBC and DanHAR. Importantly, theoretical operation count did not map monotonically to CPU latency: C3CNN had fewer FLOPs than ACESNet but required 3.776 ms per window, while TASKED had more FLOPs but was faster. This confirms the need to report both operation counts and on-device measurements rather than using parameter count or FLOPs alone as a proxy for deployment efficiency.

## 5. Discussion

This section interprets the results and states the limits of the evidence. We discuss class-specific errors, cross-subject generalization, sensor and placement variability, and edge-device deployment rather than treating overall accuracy as sufficient evidence.

### 5.1. Confusion Matrix Analysis

The confusion matrices are reported as five-seed mean row-normalized percentages rather than raw counts, which makes class-wise recall directly comparable across activities and avoids selecting one favorable seed. For readability, the figures use enlarged axis labels and cell annotations.

**Figure 7:**
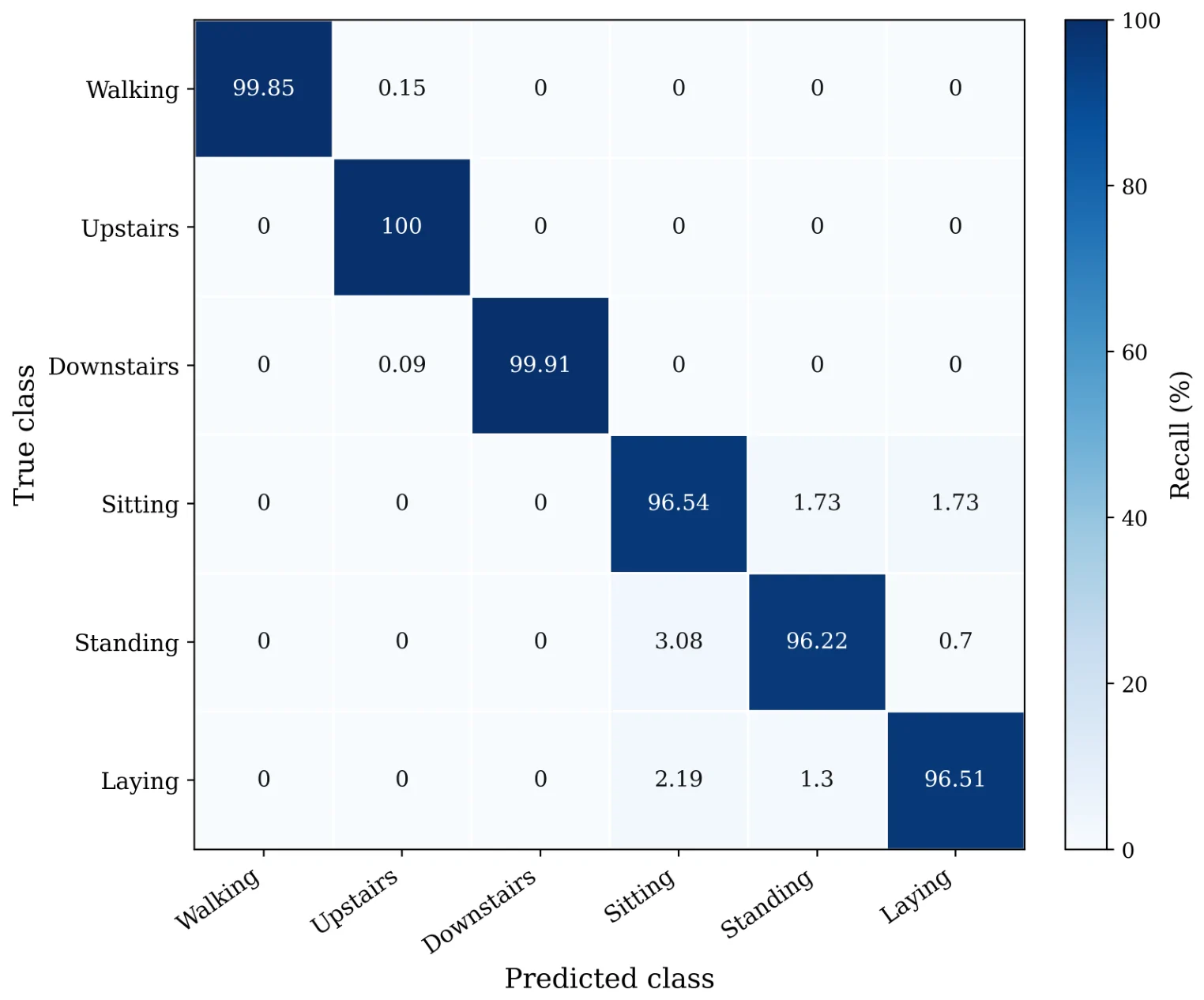
UCI-HAR confusion matrix.

On UCI-HAR (Figure 7), the three locomotion classes are almost perfectly separated: Walking, Walking Upstairs and Walking Downstairs have mean class recalls of 99.85%, 100.00% and 99.91%, respectively. The residual errors are concentrated among the static postures. In particular, 3.08% of Standing windows are classified as Sitting, while 2.19% of Laying windows are classified as Sitting. This pattern is more consistent with similarity among low-motion postures than with a failure on dynamic activities.

**Figure 8:**
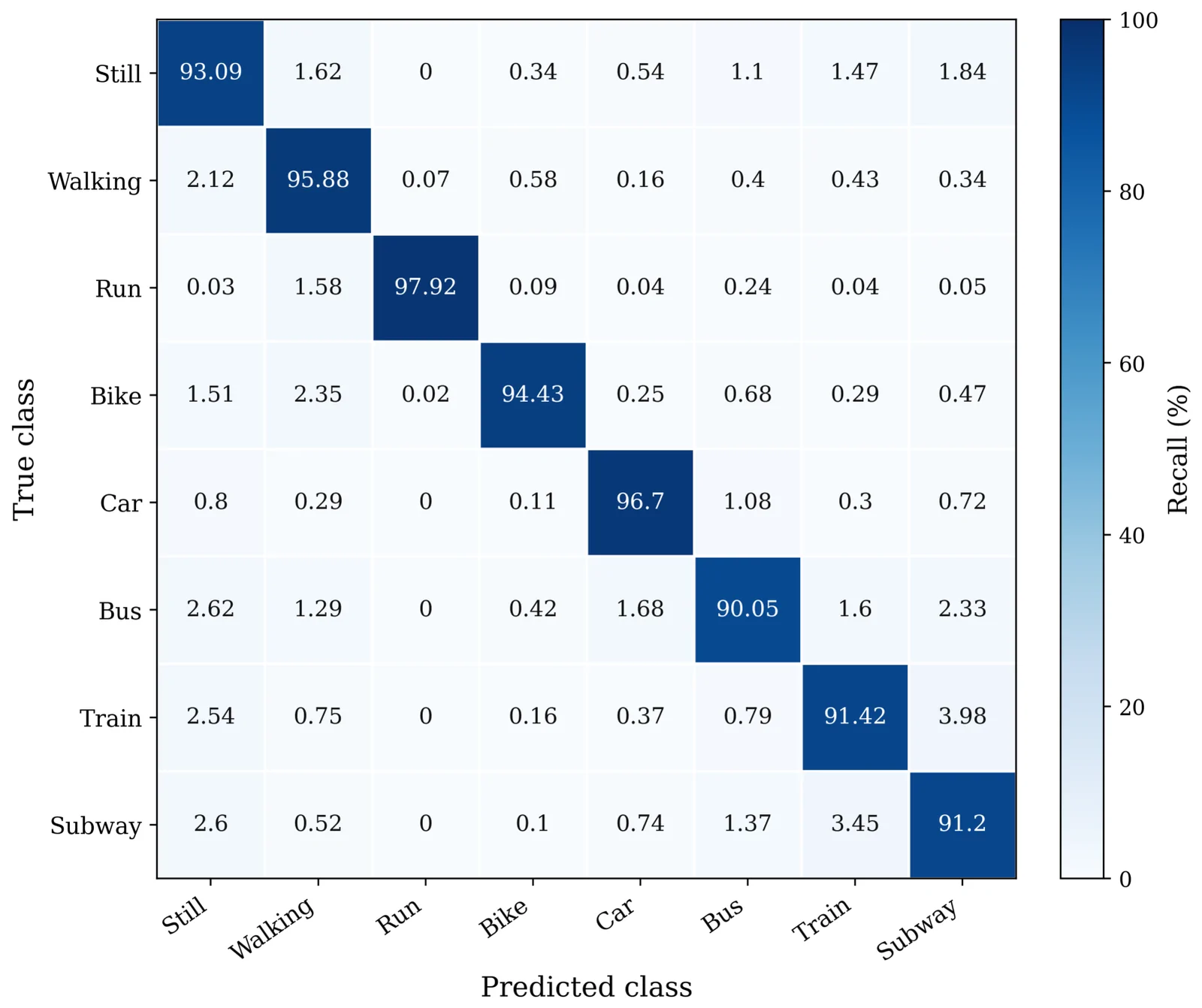
SHL confusion matrix.

SHL is more challenging because several transportation classes share low-frequency body motion and vehicle vibration. Figure 8 shows mean recalls of 97.92% for Run, 96.69% for Car and 95.90% for Walking, whereas Bus, Train and Subway obtain 90.05%, 91.42% and 91.20%, respectively. The most visible pairwise ambiguity is Train → Subway (3.98%) and Subway → Train (3.45%). Bus also shows a 2.62% confusion with Still. These results motivate the more cautious discussion of transportation-mode ambiguity in Section 5.2.

### 5.2. Error Analysis and Limitations

The ablation in Table 7 separates the principal configurable contributions of adaptive kernel selection, kernel channel attention and the parallel CNN branch. The fixed-kernel control is close in capacity to the full model (0.2609M versus 0.2623M parameters), so its lower Macro-F1 on both SHL and UCI-HAR provides evidence that the gain is not solely a parameter-count effect. Cross-scale recalibration is retained as part of the Scale-fusion implementation; because it is not independently ablated here, no standalone accuracy gain is attributed to that operation.

On SHL, the remaining confusion between Train and Subway is physically plausible because both are rail-based modes with overlapping vibration spectra and similar periods of low body motion. We regard this as an unresolved challenge. The present experiments also did not systematically vary device placement, induce missing channels, alter sampling rates, or inject controlled sensor noise. These factors can materially shift the input distribution and should be evaluated in dedicated robustness studies.

The principal benchmark used a fixed stratified window-level split. That protocol is useful for reproducible model comparison but can overestimate cross-user generalization when windows from the same participant appear in more than one subset. For this reason, we report a separate subject-independent verification in Table 8 and explicitly distinguish the two protocols. Further cross-subject and cross-device validation remains necessary, particularly for REALWORLD and SHL where wearable placement and individual motion style can vary substantially.

Finally, deployment efficiency depends on the runtime, thread configuration, precision and hardware backend rather than parameter count alone. Table 9 therefore reports a unified ONNX Runtime benchmark on a Raspberry Pi 5. The current study evaluated model inference per window and did not include sensor acquisition, operating-system scheduling, preprocessing I/O or end-to-end battery consumption. These are practical constraints for a complete wearable system and are left for future deployment studies.

For completeness, Table 10 summarizes the five-seed mean class-wise recall values discussed above.

## 6. Conclusions

This paper presented ACESNet, a three-stage convolutional architecture for six-channel inertial SHAR that combined input-dependent temporal kernel selection with lightweight multi-scale context mixing. Under the fixed five-seed window-level benchmark, ACESNet achieved 95.37%, 99.33%, 98.01% and 93.49% mean Accuracy on REALWORLD, MotionSense, UCI-HAR and SHL, respectively, with corresponding Macro-F1 scores of 95.59%, 99.12%, 98.16% and 94.03%. The component-wise ablation showed that removing the parallel CNN branch or replacing adaptive kernels with fixed kernels consistently reduced Macro-F1, while the adaptive-kernel entropy of 0.9114 provided evidence against severe single-branch collapse and was not interpreted as evidence of strong kernel selectivity.

The separate official subject-independent UCI-HAR experiment obtained 94.01 ± 0.84% Accuracy, 94.11 ± 0.88% Macro-F1 and 94.21 ± 0.84% Balanced Accuracy, quantifying the expected reduction when participant overlap was removed. The deployment study further showed that ACESNet required 8.731 MFLOPs and 1.606 ± 0.014 ms per 128-sample window on a one-thread Raspberry Pi 5 ONNX Runtime benchmark, providing a strong practical performance–efficiency trade-off without claiming the lowest latency among all evaluated models.

Several limitations remain. The principal comparison used a window-level split and therefore should not be interpreted as a fully cross-subject benchmark. The study also did not establish robustness to controlled sensor noise, missing channels, arbitrary wearable placement, heterogeneous modalities, energy consumption or MCU deployment. Broader subject-disjoint, cross-device and robustness evaluations are therefore important directions for future work.

## Figures and Tables

**Table 1:** Stage definitions of ACESNet.

Stage	$C_{\mathrm{in}}\times T_{\mathrm{in}}$	$C_{\mathrm{out}}\times T_{\mathrm{out}}$	Feature Abstraction Objective	Core Module
Patch embedding	$C_{in}\times T$	16×T/2	Channel mixing and temporal projection	Full Conv1D, k=7, stride 2
1	16×T/2	16×T/2	Local multi-scale temporal extraction	Adaptive Kernel Encoder ×2
2	16×T/2	32×T/4	Mid-range temporal aggregation	Adaptive Kernel Encoder ×2
3	32×T/4	64×T/8	High-level temporal/context abstraction	LN → Conv1D 32→64, k=2, s=2 → BN → GELU; CNN-Enhanced Scale-fusion ×6

**Table 2:** Effective receptive field.

Location	Cumulative Receptive Field (Samples)	Duration at 50 Hz
After Stage 1	31	0.62 s
After Stage 2	79	1.58 s
After Stage 3	>128 (full-window support)	2.56 s at 50 Hz; 1.28 s at 100 Hz

**Table 3:** Complexity comparison.

Mechanism	Principal Context-Mixing Term	Explicit Global Matrix
Token self-attention	$O(T^{2}C)$	T×T
SDTA/transposed attention	$O(C^{2}T)$	C×C
MSCF depthwise Scale-fusion path	$O(TC\sum_{k\in K_{s}}k)$	None
CNN-enhanced context mixer	$O\;\left(T[C\sum k+C^{2}]\right)$	None

**Table 4:** Dataset inputs and processing performed by the common experimental runner.

Item	REALWORLD	MotionSense	UCI-HAR	SHL
Subjects in source dataset	15	24	30	3
Sampling frequency (Hz)	50	50	50	100
Channels used	Acc(3)+Gyro(3)	Acc(3)+Gyro(3)	Acc(3)+Gyro(3)	Acc(3)+Gyro(3)
Input window size *T*	128	128	128	128
Additional windowing/stride/overlap	None	None	None	None
Post-load resampling/filtering/imputation	None	None	None	None
Post-load normalization/statistics fitting	None	None	None	None
Location-specific remapping in runner	None	None	None	None
Activities	8	6	6	8
Main split	70/15/15	70/15/15	70/15/15	70/15/15
Main split level	Window	Window	Window	Window
Split seed	42	42	42	42

**Table 5:** Training settings.

Item	PyTorch Group	TensorFlow/Keras Group
Models	ACESNet, C3CNN, TCN-Attention	DanHAR, DBC, TASKED
Maximum epochs	100	100
Batch size	512	64
Optimizer	AdamW	Adam
Initial learning rate	$1\times 10^{-3}$	$1\times 10^{-3}$
Weight decay	$1\times 10^{-4}$	Implementation default
LR control	5-epoch warm-up + cosine decay	ReduceLROnPlateau
Checkpoint criterion	Best validation weighted-F1	Best validation accuracy
Split seed	42 (fixed)	42 (fixed)
Training seeds	0, 1, 2, 3, 4	0, 1, 2, 3, 4
Main partition	Stratified window-level 70/15/15	

**Table 6:** Main results.

Panel A: REALWORLD and MotionSense				
**Model**	**REALWORLD**		**MotionSense**	
	**Acc.**	**Macro-F1**	**Acc.**	**Macro-F1**
C3CNN [41]	94.05 ± 0.09	94.31 ± 0.08	98.98 ± 0.17	98.94 ± 0.13
TCN-Attention [42]	95.08 ± 0.14	95.49 ± 0.13	99.04 ± 0.14	99.02 ± 0.13
DanHAR [43]	91.86 ± 0.08	91.93 ± 0.04	99.11 ± 0.13	98.96 ± 0.12
DBC [6]	94.71 ± 0.07	94.76 ± 0.06	99.06 ± 0.12	98.94 ± 0.11
TASKED [44]	93.21 ± 0.17	93.43 ± 0.13	98.01 ± 0.05	98.16 ± 0.07
**ACESNet (Ours)**	**95.37 ± 0.09**	**95.59 ± 0.09**	**99.33 ± 0.12**	**99.12 ± 0.15**
Panel B: UCI-HAR and SHL				
**Model**	**UCI-HAR**		**SHL**	
	**Acc.**	**Macro-F1**	**Acc.**	**Macro-F1**
C3CNN [41]	96.84 ± 0.31	97.01 ± 0.28	93.45 ± 0.16	93.94 ± 0.15
TCN-Attention [42]	97.61 ± 0.39	97.79 ± 0.35	92.79 ± 0.30	93.46 ± 0.27
DanHAR [43]	96.93 ± 0.13	97.17 ± 0.16	90.61 ± 0.15	90.82 ± 0.14
DBC [6]	97.62 ± 0.29	97.81 ± 0.27	89.63 ± 0.22	90.18 ± 0.21
TASKED [44]	94.65 ± 0.13	94.83 ± 0.12	88.67 ± 0.18	88.82 ± 0.20
**ACESNet (Ours)**	**98.01 ± 0.23**	**98.16 ± 0.21**	**93.49 ± 0.10**	**94.03 ± 0.08**

*Note: Bold values indicate the highest mean Accuracy or Macro-F1 for each dataset among the compared methods.*

**Table 7:** Ablation results.

Panel A: model configuration				
**Variant**	**Params (M)**	**Adaptive Kernel**	**Channel Attn.**	**Parallel CNN**
Conventional CNN	0.1741	–	–	–
Fixed-kernel ACESNet	0.2609	No	Yes	Yes
w/o kernel channel attention	0.2619	Yes	No	Yes
w/o parallel CNN branch	0.2512	Yes	Yes	No
**Full ACESNet**	0.2623	Yes	Yes	Yes
Panel B: recognition performance (%)				
**Variant**	**SHL Acc.**	**SHL Macro-F1**	**UCI Acc.**	**UCI Macro-F1**
Conventional CNN	91.93 ± 0.23	92.59 ± 0.23	97.78 ± 0.21	97.95 ± 0.19
Fixed-kernel ACESNet	93.41 ± 0.23	93.48 ± 0.20	97.63 ± 0.43	97.81 ± 0.40
w/o kernel channel attention	93.38 ± 0.02	93.74 ± 0.04	97.84 ± 0.21	98.00 ± 0.21
w/o parallel CNN branch	93.19 ± 0.12	93.29 ± 0.23	97.58 ± 0.62	97.76 ± 0.57
**Full ACESNet**	**93.47 ± 0.13**	**94.02 ± 0.10**	**97.97 ± 0.29**	**98.12 ± 0.26**

*Note: Bold identifies the full ACESNet configuration and its corresponding performance values in the ablation study.*

**Table 8:** Subject-independent UCI-HAR results.

Protocol	Accuracy (%)	Macro-F1 (%)	Balanced Accuracy (%)
Official subject-disjoint UCI-HAR split	94.01 ± 0.84	94.11 ± 0.88	94.21 ± 0.84

**Table 9:** Complexity and Raspberry Pi 5 results.

Model	Params (M)	FLOPs (M)	Size (MB)	Latency (ms)	P95 (ms)	Throughput (win/s)	Peak RSS (MB)
C3CNN	0.2937	**6.783**	**1.130**	3.776 ± 0.190	3.823	264.8	74.28
TCN-Attention	0.6168	156.388	2.463	8.772 ± 0.033	8.820	114.0	73.55
DanHAR	1.4055	323.877	5.350	11.127 ± 0.050	11.168	89.9	72.75
DBC	1.0334	254.085	3.926	8.538 ± 0.023	8.568	117.1	70.22
TASKED	0.4307	16.403	1.645	**1.160 ± 0.014**	**1.180**	**862.0**	**66.84**
**ACESNet**	**0.2623**	8.731	1.158	1.606 ± 0.014	1.622	622.7	68.28

*Note: Bold indicates the best value in each efficiency column; lower is better for Params, FLOPs, Size, Latency, P95 and Peak RSS, whereas higher is better for Throughput.*

**Table 10:** SHL class-wise recall.

Class	Still	Walking	Run	Bike	Car	Bus	Train	Subway
Recall (%)	93.09 ± 0.32	95.90 ± 0.24	97.92 ± 0.13	94.43 ± 0.12	96.69 ± 0.11	90.05 ± 0.65	91.42 ± 0.37	91.20 ± 0.23

## Data Availability

The datasets used in this study are publicly available. REALWORLD, MotionSense, UCI-HAR and SHL can be obtained from their respective official sources as cited in the references.

## Abbreviations

The following abbreviations are used in this manuscript:

HAR	Human Activity Recognition
SHAR	Sensor-based Human Activity Recognition
IMU	Inertial Measurement Unit
MEMS	Micro-Electro-Mechanical Systems
CNN	Convolutional Neural Network
RNN	Recurrent Neural Network
LSTM	Long Short-Term Memory
GRU	Gated Recurrent Unit
SE	Squeeze-and-Excitation
SDTA	Split Depth-wise Transposed Attention
MSCF	Multi-Scale Convolutional Fusion
GAP	Global Average Pooling
ACESNet	Adaptive CNN-Enhanced Scale-fusion Network
